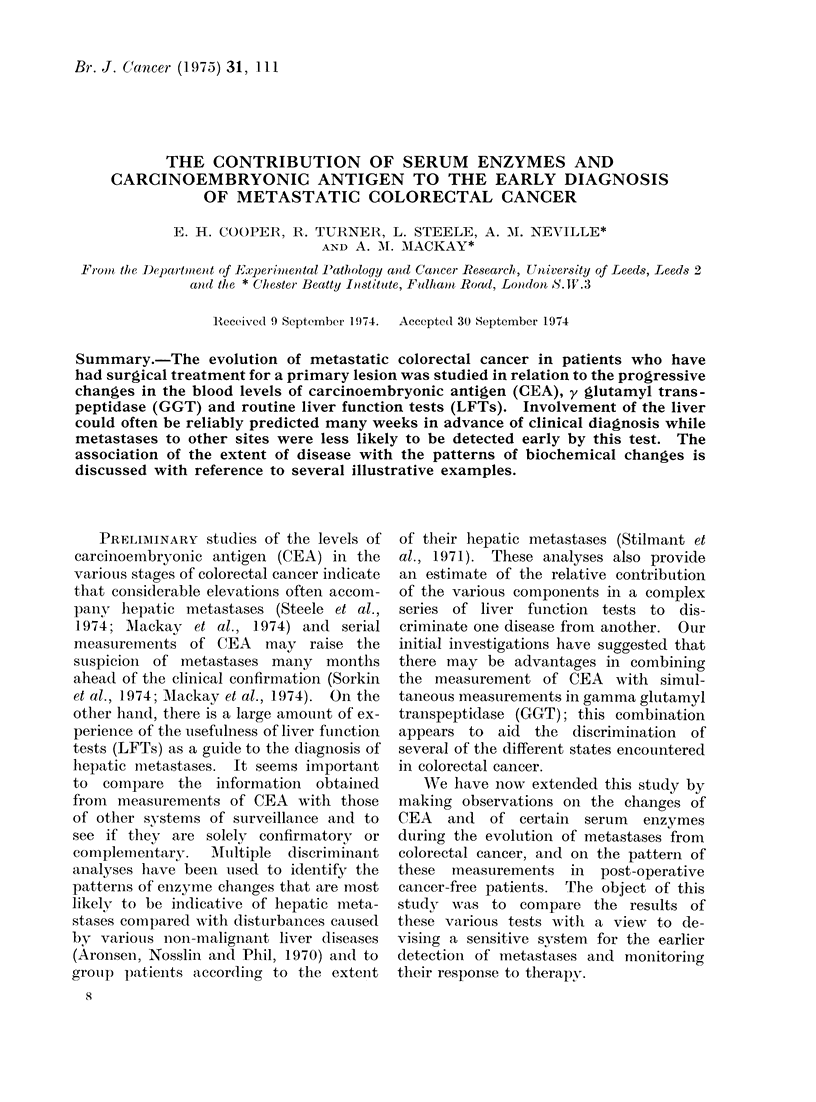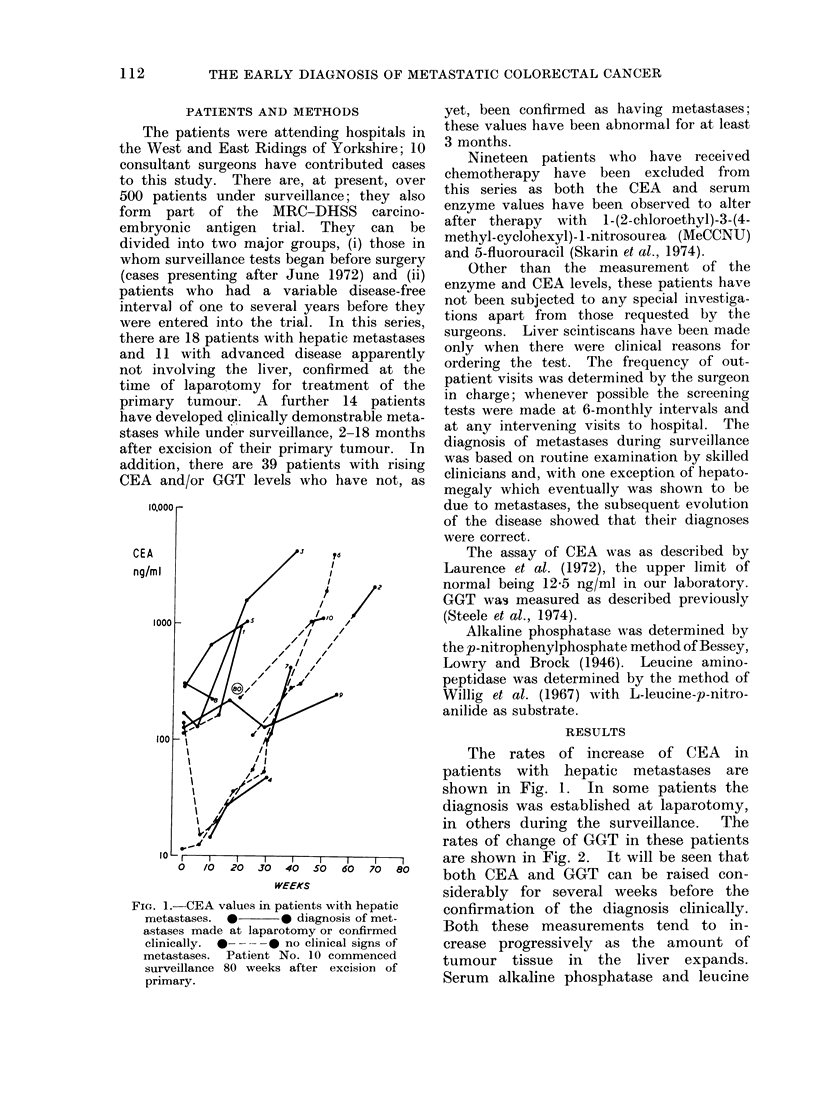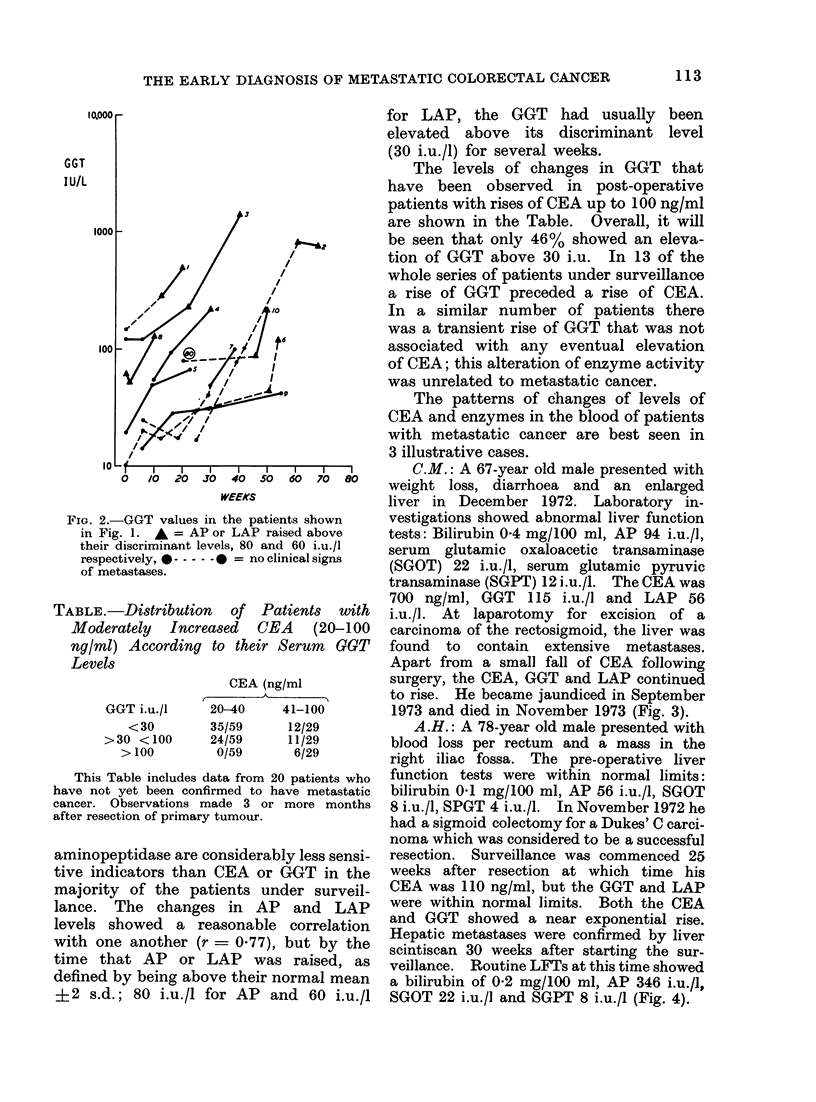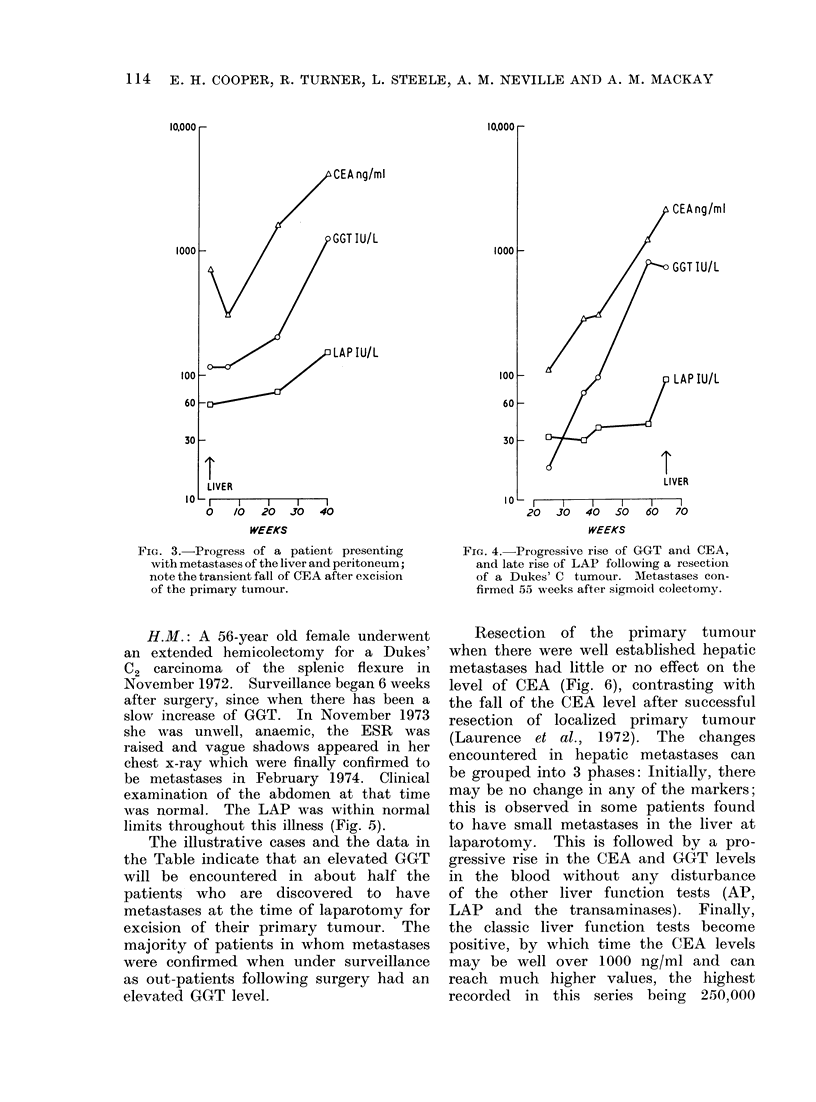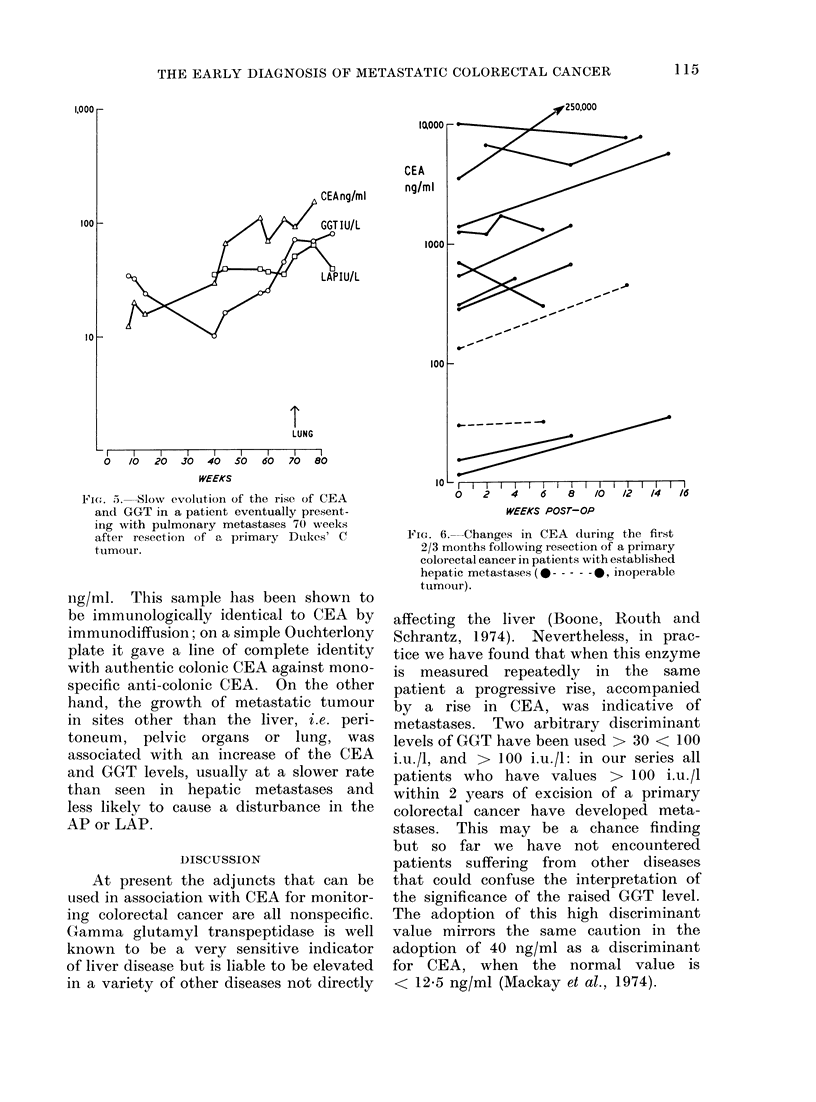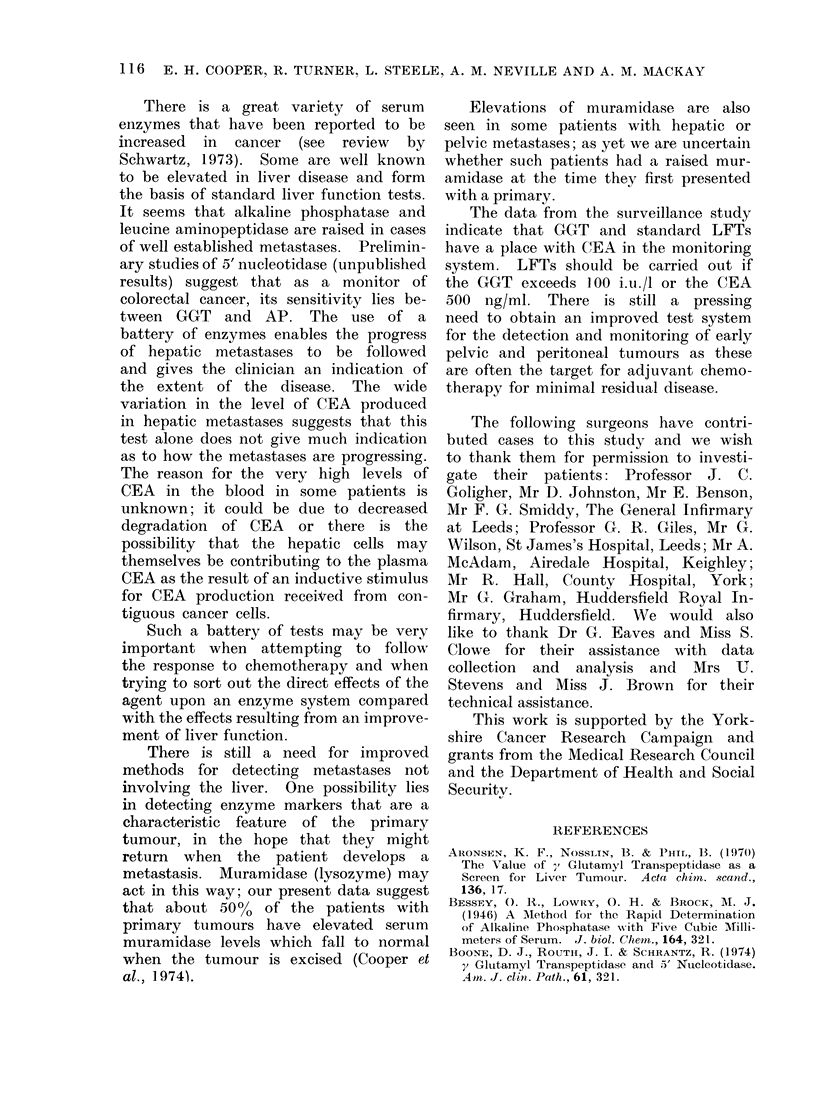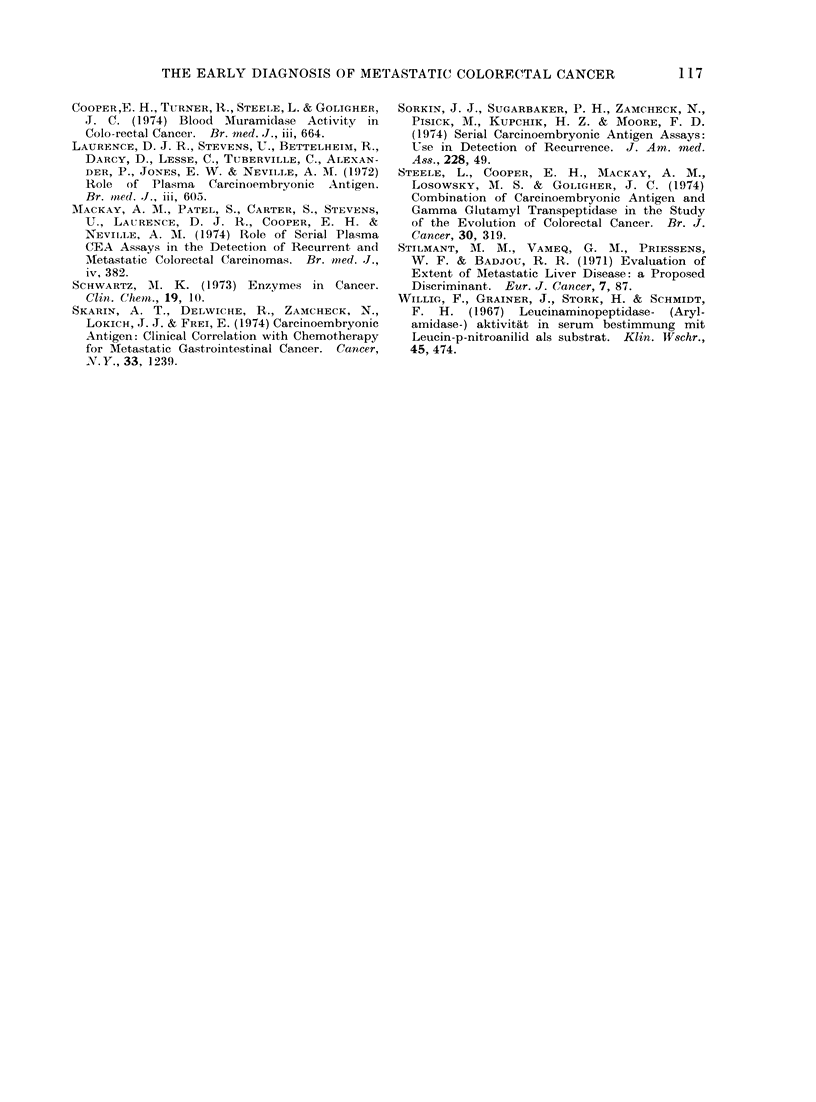# The contribution of serum enzymes and carcinoembryonic antigen to the early diagnosis of metastatic colorectal cancer.

**DOI:** 10.1038/bjc.1975.13

**Published:** 1975-01

**Authors:** E. H. Cooper, R. Turner, L. Steele, A. M. Neville, A. M. Mackay

## Abstract

The evolution of metastatic colorectal cancer in patients who have had surgical treatment for a primary lesion was studied in relation the progressive changes in the blood levels of carcinembryonic antigen (CEA), to gamma glutamyl transpeptidase (GGT) and routine liver function tests (LFTs). Involvement of the liver could ofter be reliably predicted many weeks in advance of clinical diagnosis while metastases to other sites were less likely to be detected early by this test. The association of the extent of disease with the patterns of biochemical changes is discussed with reference to several illustrative examples.


*Br. J. Cancer* (1975) **31**, 111

### THE CONTRIBUTION OF SERUM ENZYMES AND CARCINOEMBRYONIC ANTIGEN TO THE EARLY DIAGNOSIS OF METASTATIC COLORECTAL CANCER

E. H. COOPER, R. TURNER, L. STEELE, A. M. NEVILLE*
AND A. M. MACKAY*

*From the Department of Experimental Pathology and Cancer Research, University of Leeds, Leeds 2 and the * Chester Beatty Institute, Fulham Road, London S.W.3*

Received 9 September 1974. Accepted 30 September 1974

**Summary.—The evolution of metastatic colorectal cancer in patients who have had surgical treatment for a primary lesion was studied in relation to the progressive changes in the blood levels of carcinoembryonic antigen (CEA), $\gamma$ glutamyl transpeptidase (GGT) and routine liver function tests (LFTs). Involvement of the liver could often be reliably predicted many weeks in advance of clinical diagnosis while metastases to other sites were less likely to be detected early by this test. The association of the extent of disease with the patterns of biochemical changes is discussed with reference to several illustrative examples.**

PRELIMINARY studies of the levels of carcinoembryonic antigen (CEA) in the various stages of colorectal cancer indicate that considerable elevations often accompany hepatic metastases (Steele *et al.*, 1974; Mackay *et al.*, 1974) and serial measurements of CEA may raise the suspicion of metastases many months ahead of the clinical confirmation (Sorkin *et al.*, 1974; Mackay *et al.*, 1974). On the other hand, there is a large amount of experience of the usefulness of liver function tests (LFTs) as a guide to the diagnosis of hepatic metastases. It seems important to compare the information obtained from measurements of CEA with those of other systems of surveillance and to see if they are solely confirmatory or complementary. Multiple discriminant analyses have been used to identify the patterns of enzyme changes that are most likely to be indicative of hepatic metastases compared with disturbances caused by various non-malignant liver diseases (Aronsen, Nosslin and Phil, 1970) and to group patients according to the extent of their hepatic metastases (Stilmant *et al.*, 1971). These analyses also provide an estimate of the relative contribution of the various components in a complex series of liver function tests to discriminate one disease from another. Our initial investigations have suggested that there may be advantages in combining the measurement of CEA with simultaneous measurements in gamma glutamyl transpeptidase (GGT); this combination appears to aid the discrimination of several of the different states encountered in colorectal cancer.

We have now extended this study by making observations on the changes of CEA and of certain serum enzymes during the evolution of metastases from colorectal cancer, and on the pattern of these measurements in post-operative cancer-free patients. The object of this study was to compare the results of these various tests with a view to devising a sensitive system for the earlier detection of metastases and monitoring their response to therapy.

### PATIENTS AND METHODS

The patients were attending hospitals in the West and East Ridings of Yorkshire; 10 consultant surgeons have contributed cases to this study. There are, at present, over 500 patients under surveillance; they also form part of the MRC–DHSS carcinoembryonic antigen trial. They can be divided into two major groups, (i) those in whom surveillance tests began before surgery (cases presenting after June 1972) and (ii) patients who had a variable disease-free interval of one to several years before they were entered into the trial. In this series, there are 18 patients with hepatic metastases and 11 with advanced disease apparently not involving the liver, confirmed at the time of laparotomy for treatment of the primary tumour. A further 14 patients have developed clinically demonstrable metastases while under surveillance, 2–18 months after excision of their primary tumour. In addition, there are 39 patients with rising CEA and/or GGT levels who have not, as yet, been confirmed as having metastases; these values have been abnormal for at least 3 months.

FIG. 1.—CEA values in patients with hepatic metastases. ●———● diagnosis of metastases made at laparotomy or confirmed clinically. ●– – – –● no clinical signs of metastases. Patient No. 10 commenced surveillance 80 weeks after excision of primary.

Nineteen patients who have received chemotherapy have been excluded from this series as both the CEA and serum enzyme values have been observed to alter after therapy with 1-(2-chloroethyl)-3-(4-methyl-cyclohexyl)-1-nitrosourea (MeCCNU) and 5-fluorouracil (Skarin *et al.*, 1974).

Other than the measurement of the enzyme and CEA levels, these patients have not been subjected to any special investigations apart from those requested by the surgeons. Liver scintiscans have been made only when there were clinical reasons for ordering the test. The frequency of outpatient visits was determined by the surgeon in charge; whenever possible the screening tests were made at 6-monthly intervals and at any intervening visits to hospital. The diagnosis of metastases during surveillance was based on routine examination by skilled clinicians and, with one exception of hepatomegaly which eventually was shown to be due to metastases, the subsequent evolution of the disease showed that their diagnoses were correct.

The assay of CEA was as described by Laurence *et al.* (1972), the upper limit of normal being 12·5 ng/ml in our laboratory. GGT was measured as described previously (Steele *et al.*, 1974).

Alkaline phosphatase was determined by the *p*-nitrophenylphosphate method of Bessey, Lowry and Brock (1946). Leucine aminopeptidase was determined by the method of Willig *et al.* (1967) with L-leucine-*p*-nitroanilide as substrate.

### RESULTS

The rates of increase of CEA in patients with hepatic metastases are shown in Fig. 1. In some patients the diagnosis was established at laparotomy, in others during the surveillance. The rates of change of GGT in these patients are shown in Fig. 2. It will be seen that both CEA and GGT can be raised considerably for several weeks before the confirmation of the diagnosis clinically. Both these measurements tend to increase progressively as the amount of tumour tissue in the liver expands. Serum alkaline phosphatase and leucine

FIG. 2.—GGT values in the patients shown in Fig. 1. ▲ = AP or LAP raised above their discriminant levels, 80 and 60 i.u./l respectively, ●- - - - -● = no clinical signs of metastases.

TABLE.—*Distribution of Patients with Moderately Increased CEA (20–100 ng/ml) According to their Serum GGT Levels*

| GGT i.u./l | CEA (ng/ml 20–40 | 41–100 |
|---|---|---|
| <30 | 35/59 | 12/29 |
| >30 <100 | 24/59 | 11/29 |
| >100 | 0/59 | 6/29 |

This Table includes data from 20 patients who have not yet been confirmed to have metastatic cancer. Observations made 3 or more months after resection of primary tumour.

aminopeptidase are considerably less sensitive indicators than CEA or GGT in the majority of the patients under surveillance. The changes in AP and LAP levels showed a reasonable correlation with one another ($r = 0{\cdot}77$), but by the time that AP or LAP was raised, as defined by being above their normal mean ±2 s.d.; 80 i.u./l for AP and 60 i.u./l for LAP, the GGT had usually been elevated above its discriminant level (30 i.u./l) for several weeks.

The levels of changes in GGT that have been observed in post-operative patients with rises of CEA up to 100 ng/ml are shown in the Table. Overall, it will be seen that only 46% showed an elevation of GGT above 30 i.u. In 13 of the whole series of patients under surveillance a rise of GGT preceded a rise of CEA. In a similar number of patients there was a transient rise of GGT that was not associated with any eventual elevation of CEA; this alteration of enzyme activity was unrelated to metastatic cancer.

The patterns of changes of levels of CEA and enzymes in the blood of patients with metastatic cancer are best seen in 3 illustrative cases.

*C.M.*: A 67-year old male presented with weight loss, diarrhoea and an enlarged liver in December 1972. Laboratory investigations showed abnormal liver function tests: Bilirubin 0·4 mg/100 ml, AP 94 i.u./l, serum glutamic oxaloacetic transaminase (SGOT) 22 i.u./l, serum glutamic pyruvic transaminase (SGPT) 12 i.u./l. The CEA was 700 ng/ml, GGT 115 i.u./l and LAP 56 i.u./l. At laparotomy for excision of a carcinoma of the rectosigmoid, the liver was found to contain extensive metastases. Apart from a small fall of CEA following surgery, the CEA, GGT and LAP continued to rise. He became jaundiced in September 1973 and died in November 1973 (Fig. 3).

*A.H.*: A 78-year old male presented with blood loss per rectum and a mass in the right iliac fossa. The pre-operative liver function tests were within normal limits: bilirubin 0·1 mg/100 ml, AP 56 i.u./l, SGOT 8 i.u./l, SPGT 4 i.u./l. In November 1972 he had a sigmoid colectomy for a Dukes' C carcinoma which was considered to be a successful resection. Surveillance was commenced 25 weeks after resection at which time his CEA was 110 ng/ml, but the GGT and LAP were within normal limits. Both the CEA and GGT showed a near exponential rise. Hepatic metastases were confirmed by liver scintiscan 30 weeks after starting the surveillance. Routine LFTs at this time showed a bilirubin of 0·2 mg/100 ml, AP 346 i.u./l, SGOT 22 i.u./l and SGPT 8 i.u./l (Fig. 4).

FIG. 3.—Progress of a patient presenting with metastases of the liver and peritoneum; note the transient fall of CEA after excision of the primary tumour.

FIG. 4.—Progressive rise of GGT and CEA, and late rise of LAP following a resection of a Dukes' C tumour. Metastases confirmed 55 weeks after sigmoid colectomy.

*H.M.*: A 56-year old female underwent an extended hemicolectomy for a Dukes' $C_2$ carcinoma of the splenic flexure in November 1972. Surveillance began 6 weeks after surgery, since when there has been a slow increase of GGT. In November 1973 she was unwell, anaemic, the ESR was raised and vague shadows appeared in her chest x-ray which were finally confirmed to be metastases in February 1974. Clinical examination of the abdomen at that time was normal. The LAP was within normal limits throughout this illness (Fig. 5).

The illustrative cases and the data in the Table indicate that an elevated GGT will be encountered in about half the patients who are discovered to have metastases at the time of laparotomy for excision of their primary tumour. The majority of patients in whom metastases were confirmed when under surveillance as out-patients following surgery had an elevated GGT level.

Resection of the primary tumour when there were well established hepatic metastases had little or no effect on the level of CEA (Fig. 6), contrasting with the fall of the CEA level after successful resection of localized primary tumour (Laurence *et al.*, 1972). The changes encountered in hepatic metastases can be grouped into 3 phases: Initially, there may be no change in any of the markers; this is observed in some patients found to have small metastases in the liver at laparotomy. This is followed by a progressive rise in the CEA and GGT levels in the blood without any disturbance of the other liver function tests (AP, LAP and the transaminases). Finally, the classic liver function tests become positive, by which time the CEA levels may be well over 1000 ng/ml and can reach much higher values, the highest recorded in this series being 250,000

FIG. 5.—Slow evolution of the rise of CEA and GGT in a patient eventually presenting with pulmonary metastases 70 weeks after resection of a primary Dukes' C tumour.

FIG. 6.—Changes in CEA during the first 2/3 months following resection of a primary colorectal cancer in patients with established hepatic metastases (●- - - - -●, inoperable tumour).

ng/ml. This sample has been shown to be immunologically identical to CEA by immunodiffusion; on a simple Ouchterlony plate it gave a line of complete identity with authentic colonic CEA against monospecific anti-colonic CEA. On the other hand, the growth of metastatic tumour in sites other than the liver, *i.e.* peritoneum, pelvic organs or lung, was associated with an increase of the CEA and GGT levels, usually at a slower rate than seen in hepatic metastases and less likely to cause a disturbance in the AP or LAP.

### DISCUSSION

At present the adjuncts that can be used in association with CEA for monitoring colorectal cancer are all nonspecific. Gamma glutamyl transpeptidase is well known to be a very sensitive indicator of liver disease but is liable to be elevated in a variety of other diseases not directly affecting the liver (Boone, Routh and Schrantz, 1974). Nevertheless, in practice we have found that when this enzyme is measured repeatedly in the same patient a progressive rise, accompanied by a rise in CEA, was indicative of metastases. Two arbitrary discriminant levels of GGT have been used $> 30 < 100$ i.u./l, and $> 100$ i.u./l: in our series all patients who have values $> 100$ i.u./l within 2 years of excision of a primary colorectal cancer have developed metastases. This may be a chance finding but so far we have not encountered patients suffering from other diseases that could confuse the interpretation of the significance of the raised GGT level. The adoption of this high discriminant value mirrors the same caution in the adoption of 40 ng/ml as a discriminant for CEA, when the normal value is $< 12{\cdot}5$ ng/ml (Mackay *et al.*, 1974).

There is a great variety of serum enzymes that have been reported to be increased in cancer (see review by Schwartz, 1973). Some are well known to be elevated in liver disease and form the basis of standard liver function tests. It seems that alkaline phosphatase and leucine aminopeptidase are raised in cases of well established metastases. Preliminary studies of 5′ nucleotidase (unpublished results) suggest that as a monitor of colorectal cancer, its sensitivity lies between GGT and AP. The use of a battery of enzymes enables the progress of hepatic metastases to be followed and gives the clinician an indication of the extent of the disease. The wide variation in the level of CEA produced in hepatic metastases suggests that this test alone does not give much indication as to how the metastases are progressing. The reason for the very high levels of CEA in the blood in some patients is unknown; it could be due to decreased degradation of CEA or there is the possibility that the hepatic cells may themselves be contributing to the plasma CEA as the result of an inductive stimulus for CEA production received from contiguous cancer cells.

Such a battery of tests may be very important when attempting to follow the response to chemotherapy and when trying to sort out the direct effects of the agent upon an enzyme system compared with the effects resulting from an improvement of liver function.

There is still a need for improved methods for detecting metastases not involving the liver. One possibility lies in detecting enzyme markers that are a characteristic feature of the primary tumour, in the hope that they might return when the patient develops a metastasis. Muramidase (lysozyme) may act in this way; our present data suggest that about 50% of the patients with primary tumours have elevated serum muramidase levels which fall to normal when the tumour is excised (Cooper *et al.*, 1974).

Elevations of muramidase are also seen in some patients with hepatic or pelvic metastases; as yet we are uncertain whether such patients had a raised muramidase at the time they first presented with a primary.

The data from the surveillance study indicate that GGT and standard LFTs have a place with CEA in the monitoring system. LFTs should be carried out if the GGT exceeds 100 i.u./l or the CEA 500 ng/ml. There is still a pressing need to obtain an improved test system for the detection and monitoring of early pelvic and peritoneal tumours as these are often the target for adjuvant chemotherapy for minimal residual disease.

The following surgeons have contributed cases to this study and we wish to thank them for permission to investigate their patients: Professor J. C. Goligher, Mr D. Johnston, Mr E. Benson, Mr F. G. Smiddy, The General Infirmary at Leeds; Professor G. R. Giles, Mr G. Wilson, St James's Hospital, Leeds; Mr A. McAdam, Airedale Hospital, Keighley; Mr R. Hall, County Hospital, York; Mr G. Graham, Huddersfield Royal Infirmary, Huddersfield. We would also like to thank Dr G. Eaves and Miss S. Clowe for their assistance with data collection and analysis and Mrs U. Stevens and Miss J. Brown for their technical assistance.

This work is supported by the Yorkshire Cancer Research Campaign and grants from the Medical Research Council and the Department of Health and Social Security.

### REFERENCES

Aronsen, K. F., Nosslin, B. & Phil, B. (1970) The Value of γ Glutamyl Transpeptidase as a Screen for Liver Tumour. *Acta chim. scand.*, **136**, 17.

Bessey, O. R., Lowry, O. H. & Brock, M. J. (1946) A Method for the Rapid Determination of Alkaline Phosphatase with Five Cubic Millimeters of Serum. *J. biol. Chem.*, **164**, 321.

Boone, D. J., Routh, J. I. & Schrantz, R. (1974) γ Glutamyl Transpeptidase and 5′ Nucleotidase. *Am. J. clin. Path.*, **61**, 321.

COOPER, E. H., TURNER, R., STEELE, L. & GOLIGHER, J. C. (1974) Blood Muramidase Activity in Colo-rectal Cancer. *Br. med. J.*, iii, 664.

LAURENCE, D. J. R., STEVENS, U., BETTELHEIM, R., DARCY, D., LESSE, C., TUBERVILLE, C., ALEXANDER, P., JONES, E. W. & NEVILLE, A. M. (1972) Role of Plasma Carcinoembryonic Antigen. *Br. med. J.*, iii, 605.

MACKAY, A. M., PATEL, S., CARTER, S., STEVENS, U., LAURENCE, D. J. R., COOPER, E. H. & NEVILLE, A. M. (1974) Role of Serial Plasma CEA Assays in the Detection of Recurrent and Metastatic Colorectal Carcinomas. *Br. med. J.*, iv, 382.

SCHWARTZ, M. K. (1973) Enzymes in Cancer. *Clin. Chem.*, **19**, 10.

SKARIN, A. T., DELWICHE, R., ZAMCHECK, N., LOKICH, J. J. & FREI, E. (1974) Carcinoembryonic Antigen: Clinical Correlation with Chemotherapy for Metastatic Gastrointestinal Cancer. *Cancer, N.Y.*, **33**, 1239.

SORKIN, J. J., SUGARBAKER, P. H., ZAMCHECK, N., PISICK, M., KUPCHIK, H. Z. & MOORE, F. D. (1974) Serial Carcinoembryonic Antigen Assays: Use in Detection of Recurrence. *J. Am. med. Ass.*, **228**, 49.

STEELE, L., COOPER, E. H., MACKAY, A. M., LOSOWSKY, M. S. & GOLIGHER, J. C. (1974) Combination of Carcinoembryonic Antigen and Gamma Glutamyl Transpeptidase in the Study of the Evolution of Colorectal Cancer. *Br. J. Cancer*, **30**, 319.

STILMANT, M. M., VAMEQ, G. M., PRIESSENS, W. F. & BADJOU, R. R. (1971) Evaluation of Extent of Metastatic Liver Disease: a Proposed Discriminant. *Eur. J. Cancer*, **7**, 87.

WILLIG, F., GRAINER, J., STORK, H. & SCHMIDT, F. H. (1967) Leucinaminopeptidase- (Arylamidase-) aktivität in serum bestimmung mit Leucin-p-nitroanilid als substrat. *Klin. Wschr.*, **45**, 474.